# Spectroscopy data of ceftriaxone-lysozyme interaction and computational studies

**DOI:** 10.1016/j.dib.2018.04.079

**Published:** 2018-04-30

**Authors:** Paolo Ruzza, Rosa Maria Vitale, Rohanah Hussain, Alessia Montini, Claudia Honisch, Alice Pozzebon, Charlotte S. Hughes, Barbara Biondi, Pietro Amodeo, GianPietro Sechi, Giuliano Siligardi

**Affiliations:** a Institute of Biomolecular Chemistry of CNR, Padua Unit, Padua, Italy; b Institute of Biomolecular Chemistry of CNR, Pozzuoli, Italy; c Diamond Light Source Ltd., Harwell Science and Innovation Campus, Didcot, Oxfordshire OX11 0DE, United Kingdom; d Department of Clinical, Surgery and Experimental Medicine, Medical School, University of Sassari, Sassari, Italy

## Abstract

The data article presents the results obtained from fluorescence and synchrotron radiation circular dichroism spectroscopies about the lysozyme-ceftriaxone interaction at neutral and acidic pH values as well as the computational calculations described in the accompanying research article (Ruzza et al., sub) [1].

**Specifications table**

**Table:** 

Subject area	*Chemistry*
More specific subject area	*antimicrobial compounds, protein aggregation, anti-amyloidogenic compounds*
Type of data	*Table, graph, figure*
How data was acquired	*Synchrotron radiation circular dichroism and fluorescence spectra. SRCD at Diamond Light Source, beamline B23, module B; fluorescence: Perkin-Elmer LS-50B spectrofluorimeter; DLS: Malvern Zetasizer Nano ZSP.*
Data format	*Analyzed with CDApps and Origin 9*
Experimental factors	*N/A*
Experimental features	*The potential thermal and UV stabilizing effects of ceftriaxone on HL and HEWL were monitored as complementary methods to determine ceftriaxone-lysozyme binding interactions.*
Data accessibility	*All referenced data is in the article.*

**Value of the data**

• Data enlarges the panel of available anti-amyloidogenic molecules acting on lysozyme
• Data expands the array of known amylodogenic targets of ceftriaxone, a widely-used antibiotic
• Data provides new references for characterization of anti-amyloidogenic compounds

## 1 Data

**Fig. 1:**
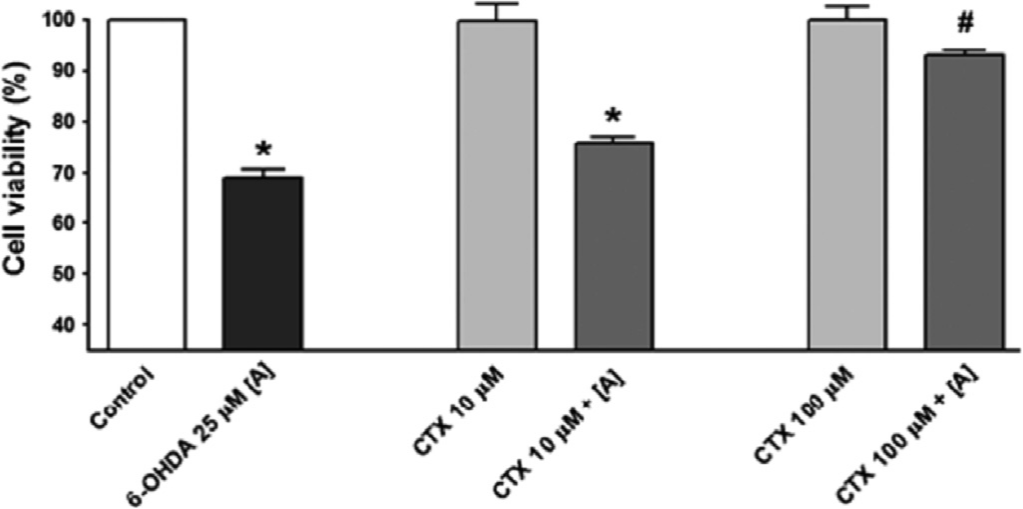
Apoptosis prevention by Ceftriazone on PC12 incubated with neurotoxin oxydopamine, 6-OHDA (redrawn from Ref. [2]).

**Fig. 2:**
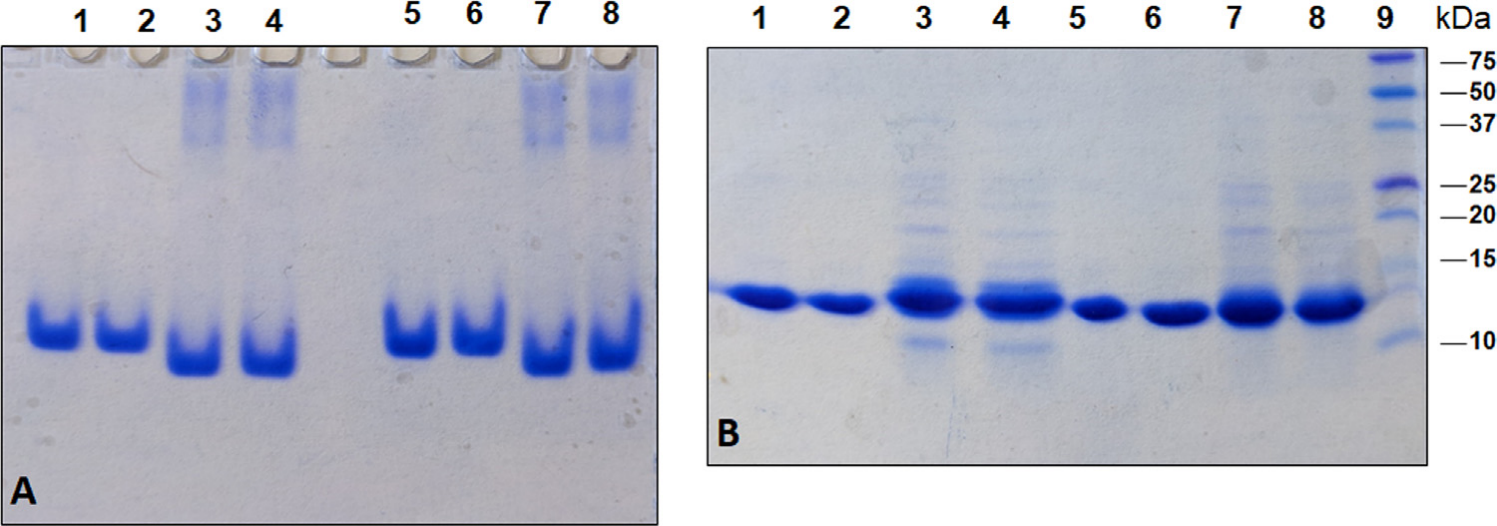
(**A**) Native PAGE of hen egg white (1, 2, 5, 6) and human (3, 4, 7, 8) lysozyme in absence (1–5) or presence of 2 M urea (5–8). Samples 1, 3, 5, 7 are dissolved in 70 mM Gly-HCl/80 mM NaCl buffer, pH 2.7; samples 2, 4, 6, 8 are dissolved in 20 mM phosphate buffer, pH 6.8. (**B**) SDS PAGE of hen egg white (1, 2, 5, 6) and human (3, 4, 7, 8) lysozyme in rid (1–5) or non rid (5–8). Samples 1, 3, 5, 7 are dissolved in 70 mM Gly-HCl/80 mM NaCl buffer, pH 2.7; samples 2, 4, 6, 8 are dissolved in 20 mM phosphate buffer, pH 6.8. The results indicated the absence of dimeric or oligomeric states of both proteins in the experimental condition. Commercial recombinant HL protein results to be contaminated.

**Fig. 3:**
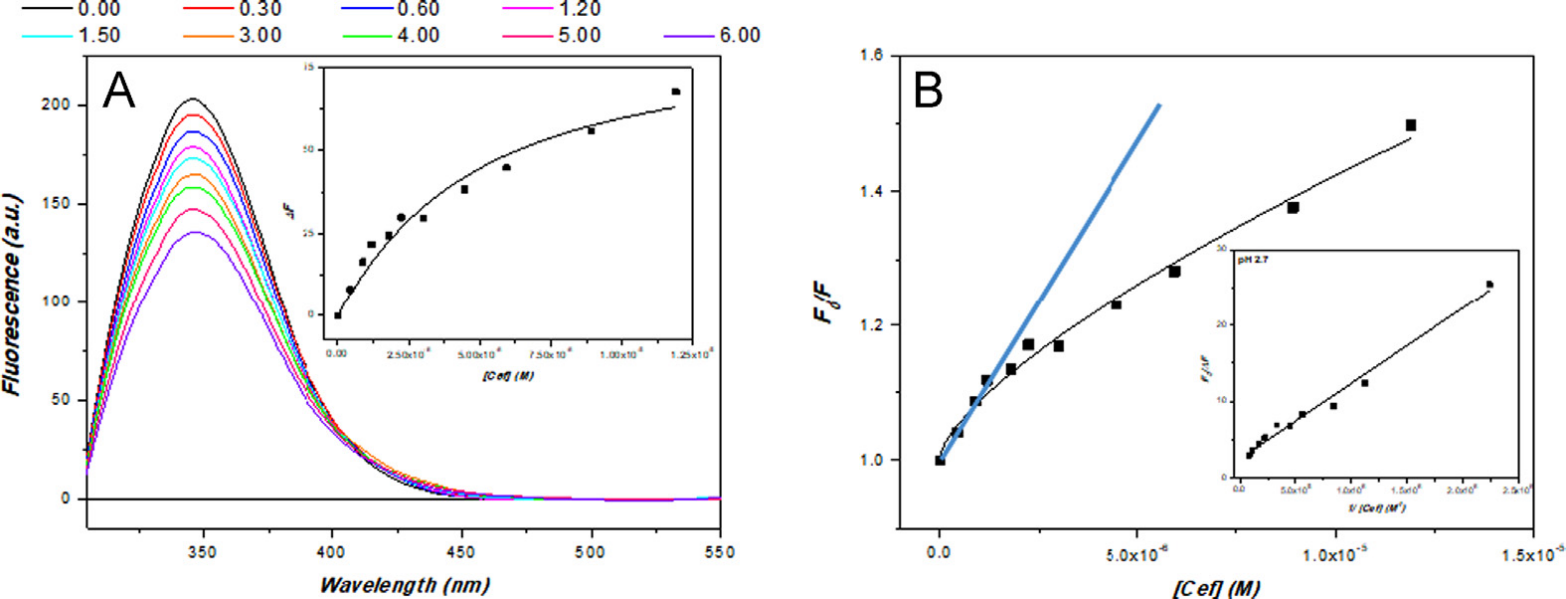
(**A**) HEWL fluorescence emission at increasing amounts of ceftriaxone. Fluorescence spectra were measured as a function of the increasing ceftriaxone/lysozyme molar ratios (indicated) in 70 mM Gly-HCl/80 mM NaCl buffer, pH 2.7, at 25 °C. Lysozyme 1.68 µM and ceftriaxone stock concentration solution 12.10 µM. Insert: plot of relative fluorescence changes versus ceftriaxone concentration. (**B**) Stern-Volmer plot of quenching of HEWL by ceftriaxone (in cyan the expected plot). Insert: modified Stern-Volmer plot to determine the accessible fraction (*f*_a_).

**Fig. 4:**
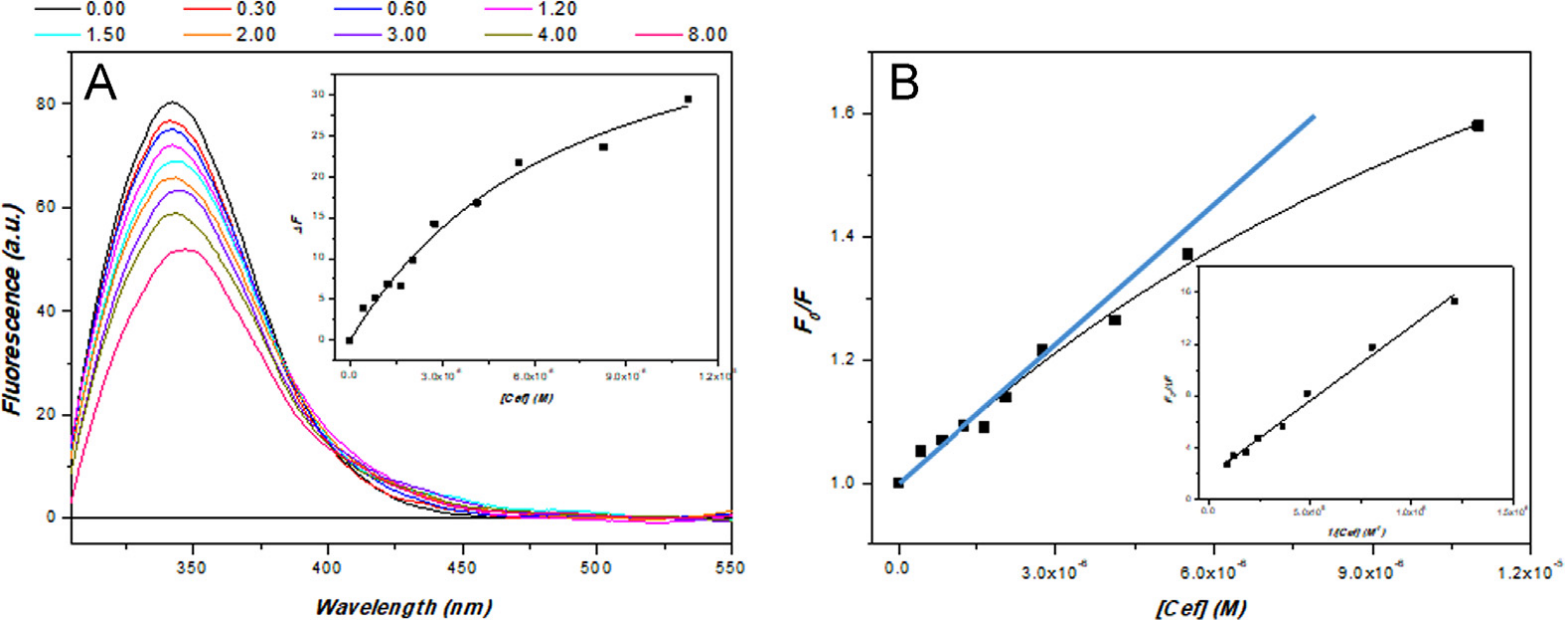
(**A**) HL fluorescence emission at increasing amounts of ceftriaxone. Fluorescence spectra were measured as a function of the increasing ceftriaxone/lysozyme molar ratios (indicated) in 20 mM phosphate buffer, pH 6.8, at 25 °C. Lysozyme 1.52 µM and ceftriaxone stock solution 11.00 µM. Insert: plot of relative fluorescence changes versus ceftriaxone concentration. (**B**) Stern-Volmer plot of quenching of HL by ceftriaxone (in cyan the expected plot). Insert: modified Stern-Volmer plot to determine the accessible fraction (*f*_a_).

**Fig. 5:**
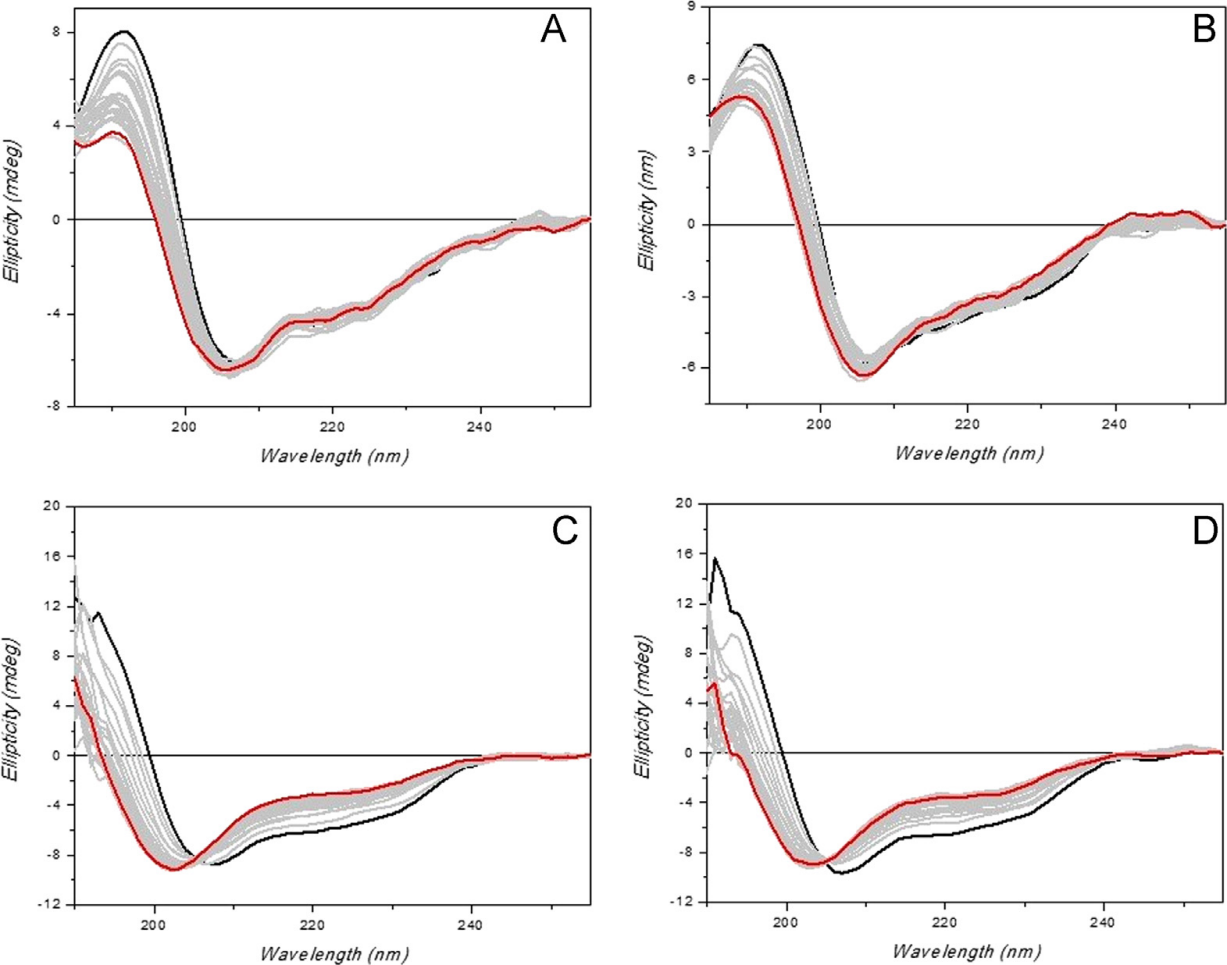
HEWL UV-denaturation assay. Twenty-five repeated consecutive SRCD scans of HEWL (0.5 mg/mL) alone (**A** and **C**) or in presence of 2 eq. of Cef (**B** and **D**). Samples were dissolved either in 20 mM phosphate buffer solution, pH 6.8 (**A** and **B**), or in 70 mM glycine-HCl/80 mM NaCl buffer, pH 2.7 (**C** and **D**). SRCD spectra were measured with B23 module B. The solid black line indicates the first scan and the solid red line the 25th scan. Integration time 1 s, 0.02 cm cylindrical cell (60 µl), monochromator bandwidth 1.8 nm.

**Fig. 6:**
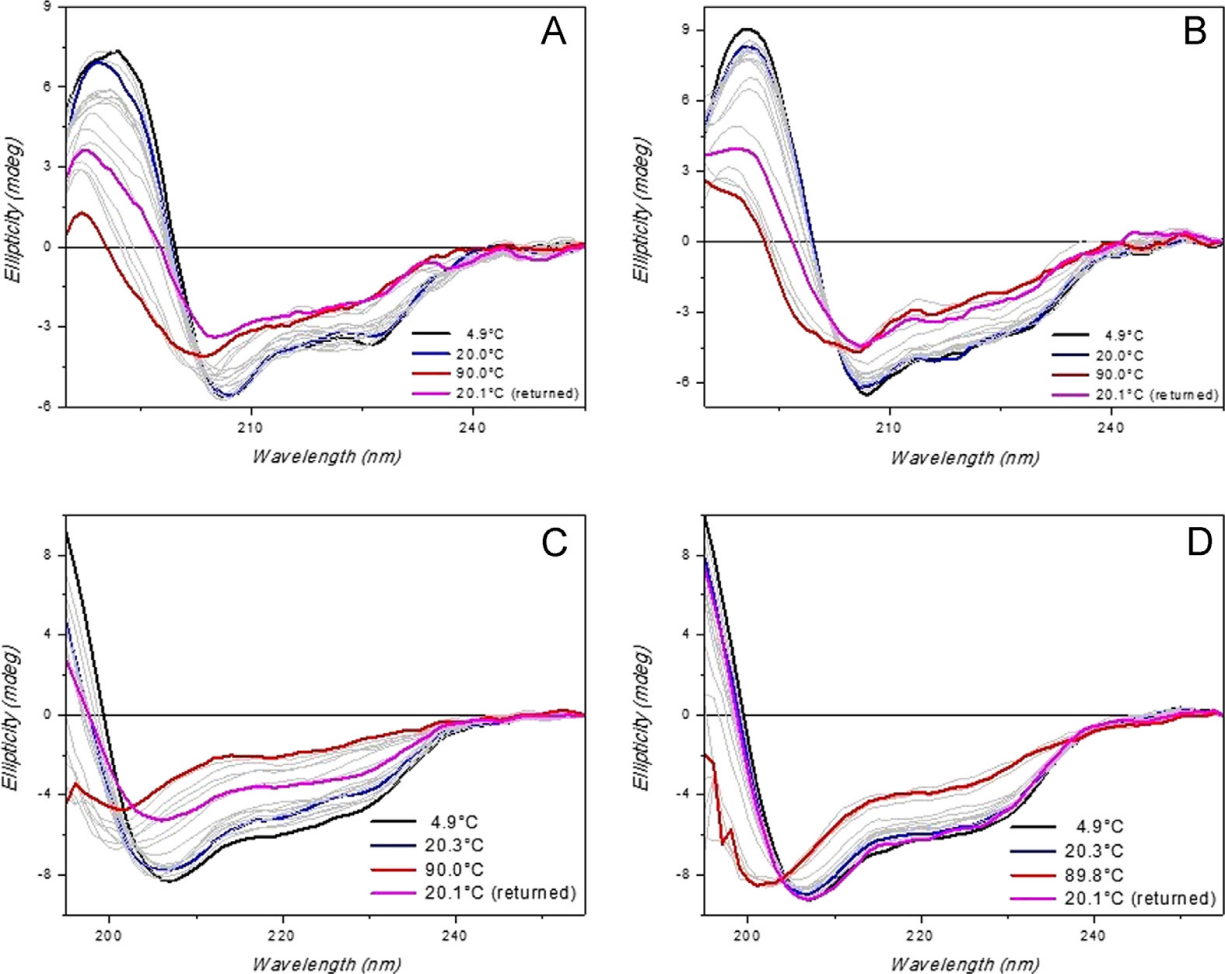
HEWL thermal denaturation assay. Far-UV SRCD spectra of HEWL (0.5 mg/mL) alone (**A** and **C**) or in presence of 2 eq. of **Cef** (**B** and **D**). Samples were dissolved either in 20 mM phosphate buffer solution, pH 6.8 (**A** and **B**), or in 70 mM glycine-HCl/80 mM NaCl buffer, pH 2.7 (**C** and **D**). SRCD spectra were measured with B23 module B at different temperatures (indicated). Integration time 1 s, 0.02 cm cylindrical cell (60 µl), monochromator bandwidth 1.2 nm.

**Fig. 7:**
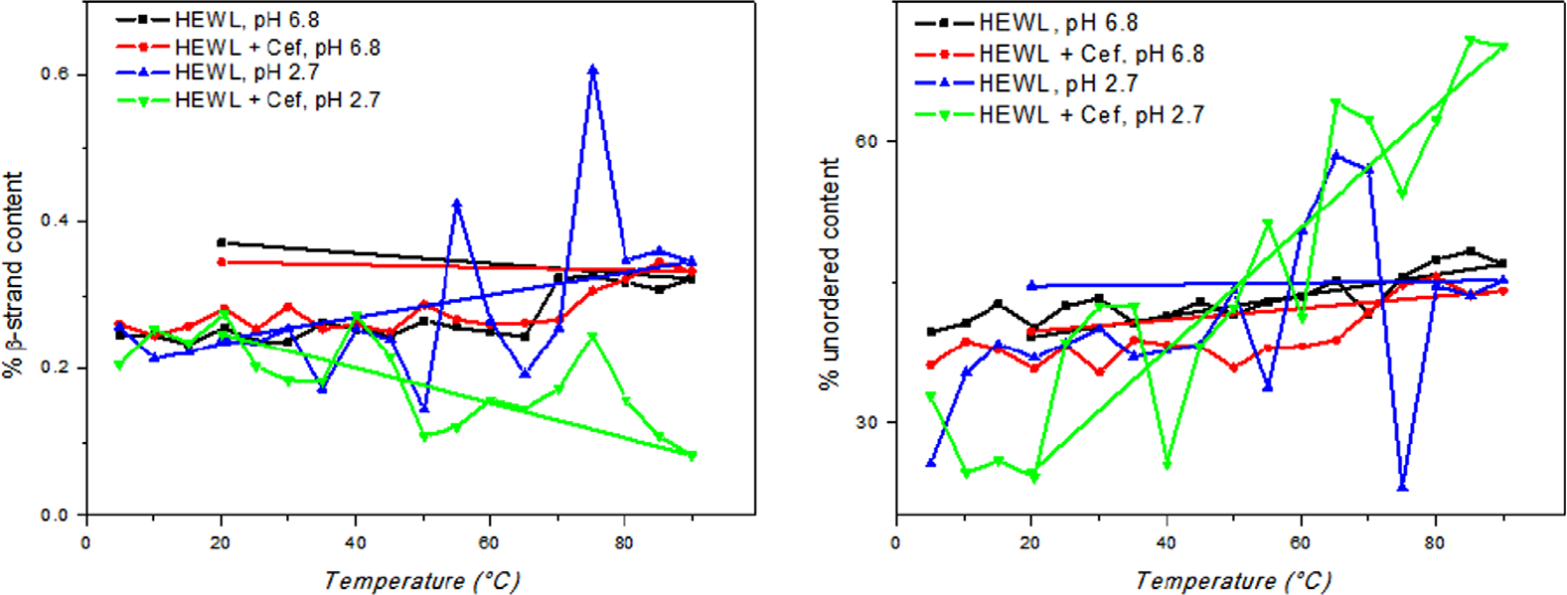
Secondary structure content versus temperature. Plot of β-strand and unordered content for the HEWL alone (black, pH 6.8; blue, pH 2.7) and in presence of 2 eq. of ceftriaxone (red, pH 6.8; magenta, pH 2.7) determined with CONTINLL [5] of CDApps [6] from SRCD data versus temperature.

**Fig. 8:**
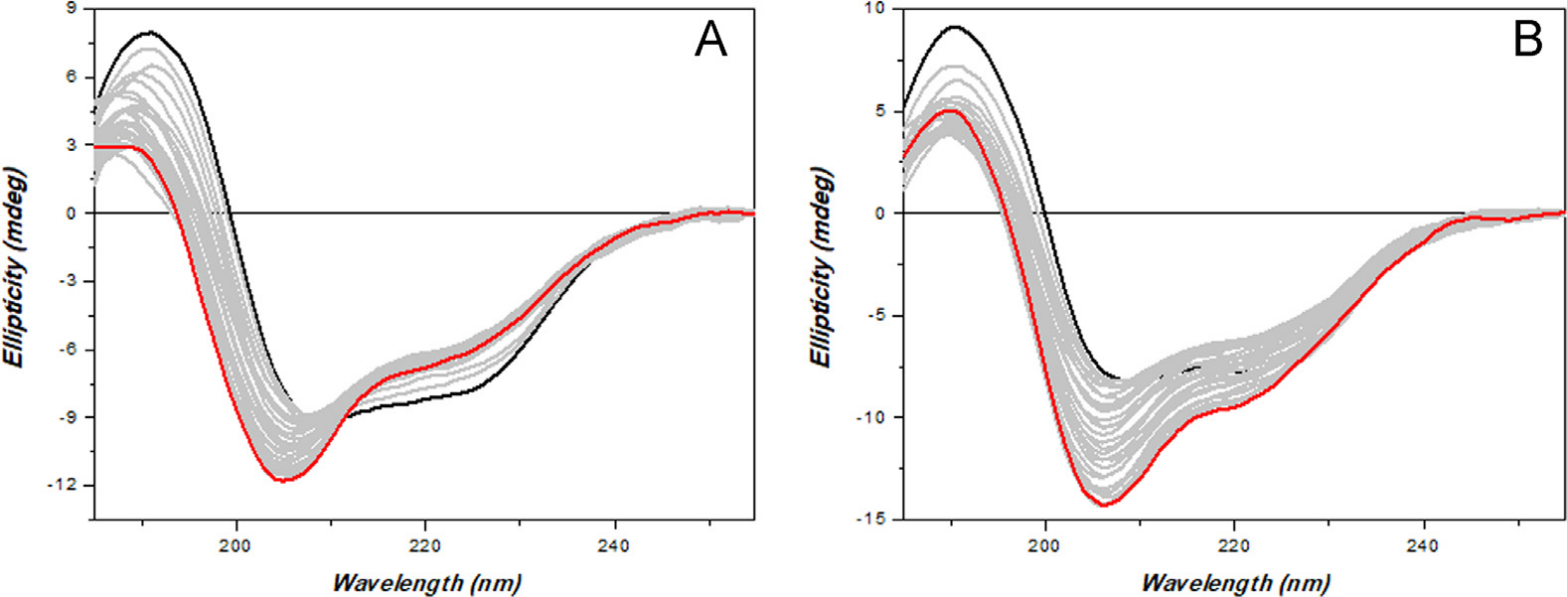
HL UV-denaturation assay. Twenty-five repeated consecutive SRCD scans of HL (0.5 mg/mL) alone (**A**) or in presence of 2 eq. of **Cef** (**B**) in 20 mM phosphate buffer solution, pH 6.8, measured with B23 module B. The solid black line indicates the first scan and the solid red line the 25th scan. Integration time 1 s, 0.02 cm cylindrical cell (60 µl), monochromator bandwidth 1.8 nm.

**Fig. 9:**
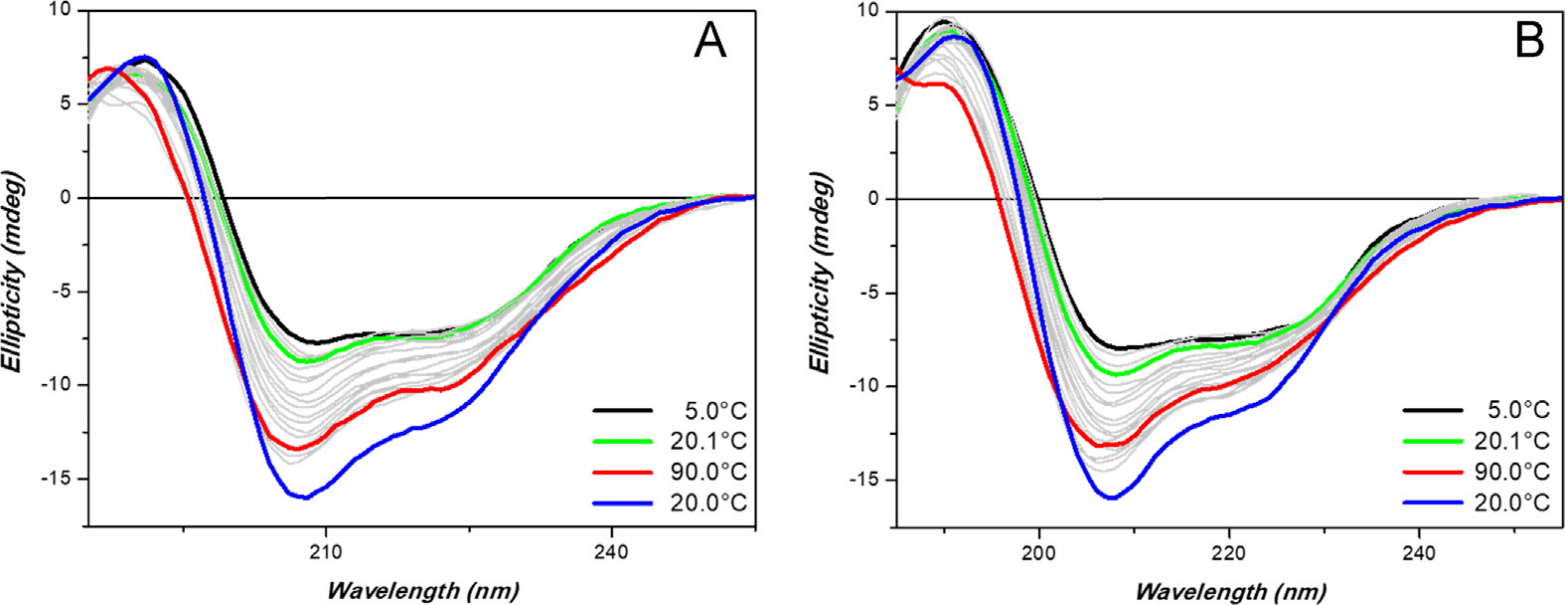
HL thermal denaturation assay. (**A** and **B**) Far-UV SRCD spectra of HL (0.5 mg/mL) alone (**A**) or in presence of 2 eq. of **Cef** (**B**) in 20 mM phosphate buffer solution, pH 6.8, measured with B23 module B at different temperatures. Integration time 1 s, 0.02 cm cylindrical cell (60 µl), monochromator bandwidth 1.2 nm.

**Fig. 10:**
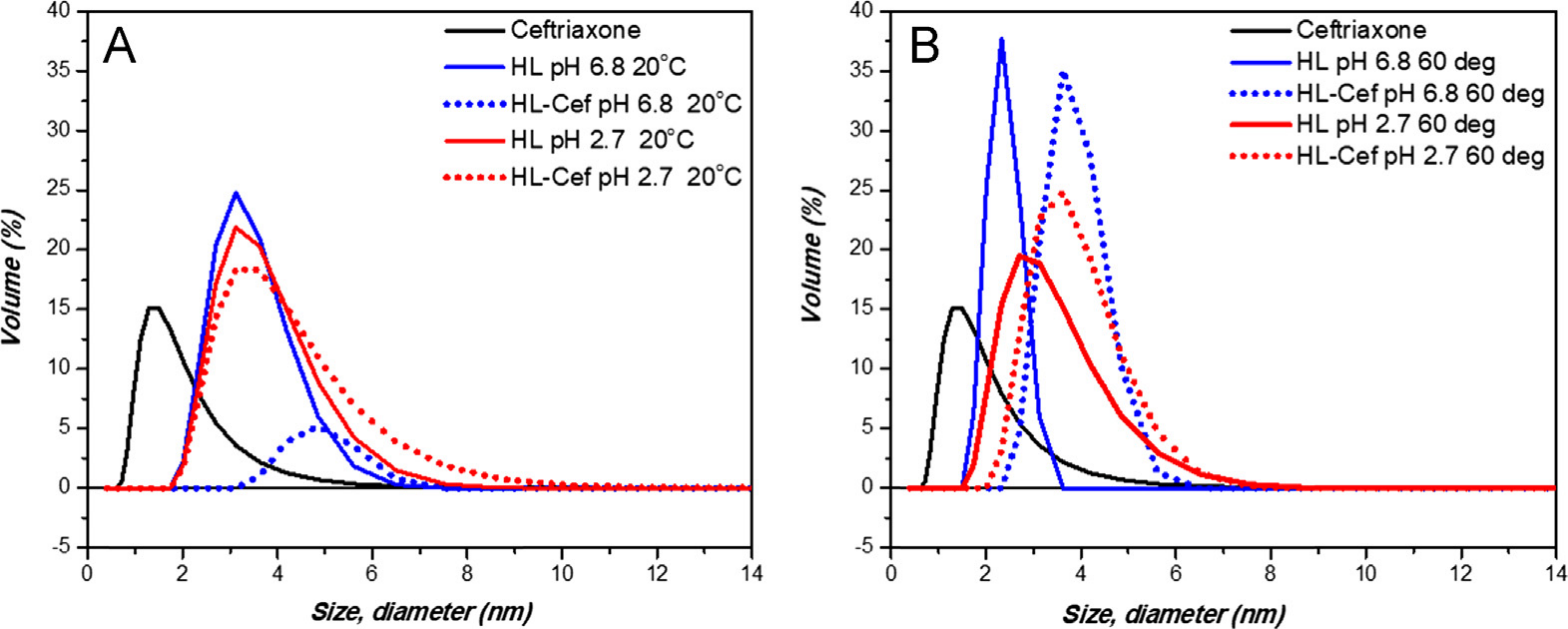
Dynamic Light Scattering analysis. Volume (%) vs. size (d.nm) for HL in the presence and absence of **Cef** at different pH values, either at 20 °C (**A**) or at 60 °C (**B**). Dynamic light scattering was recorded on a Zetasizer Nano ZSP (Malvern). Size (diameter, nm) vs. volume (%) was plotted pre- and post- thermal denaturation; and as a function of temperature (°C).

**Fig. 11:**
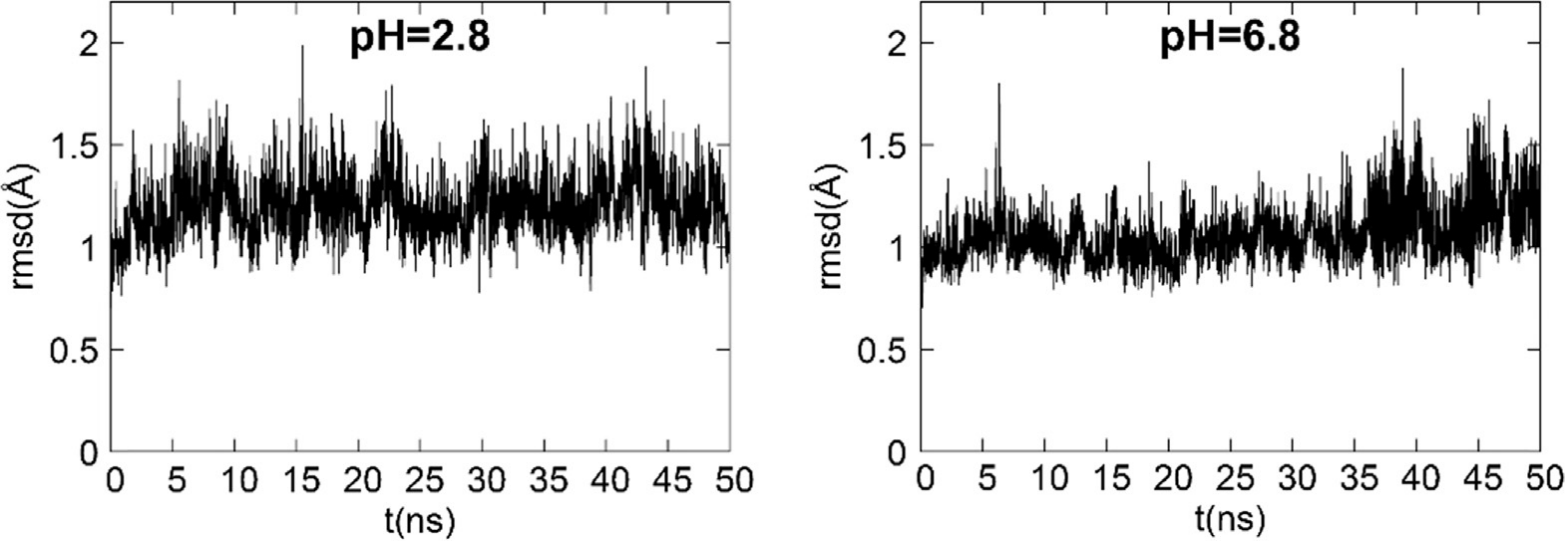
Time evolution plot of backbone atom-positional rmsd of HEWL protein over the MD simulated period (50 ns) at acidic (left) and neutral (right) pH values.

**Fig. 12:**
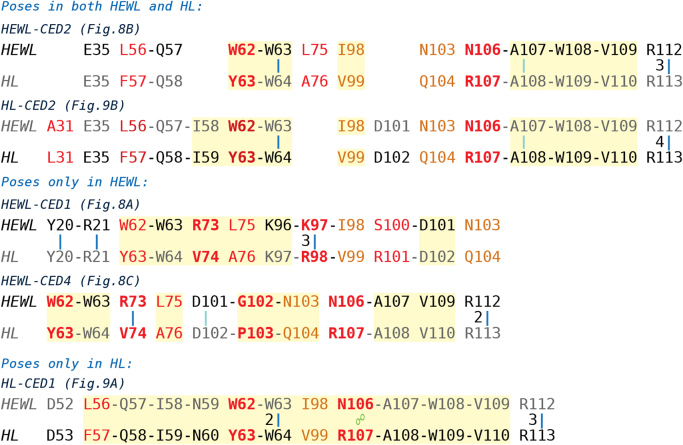
HEWL-HL sequence alignments in ligand-interaction regions of **Cef** complexes shown in Fig. 7, Fig. 8, Fig. 9. For each complex, the protein residues forming contacts with **Cef** atoms within 5 Å are shown in black, while the corresponding residues of the other protein are reported in gray. In both sequences, red bold, red and dark orange characters indicate major, non-conservative and conservative mutations, respectively. Light yellow areas enclose residues forming hydrophobic patches/pockets in which the ligand docks in the pose under examination; cyan lines, blue lines and green two connected circles show H-bonds involving protein backbone, H-bonds involving protein sidechain, and π-stacking interaction, respectively. For multiple H-bonds, the number of interactions is shown on the left of the corresponding symbol.

Evaluation of ceftriazone Ceftriaxone (**Cef**, CAS number 104376-79-6) on PC12 apoptosis using 6-OHDA cell toxicity assay (Fig. 1) was identified [2]. The influence of pH values on the monomeric or oligomeric states of lysozyme was evaluated by native PAGE (Fig. 2). Fluorescence spectroscopy measurements were used to determine the dissociation constant (*K*_d_) (Figs. 3A and 4A) using a nonlinear regression analysis [3]. Fluorophorus populations: one accessible to the quencher and the other inaccessible (see Table 1 in Ref. [1]) was determined by The Stern-Volmer analysis [4] of the Trp emission quenching (Figs. 3B and 4B). The influence of **Cef** on lysozyme stability was evaluated by both UV and thermal denaturation assays using synchrotron radiation circular dichroism (Fig. 5, Fig. 6, Fig. 7, Fig. 8, Fig. 9). Hydrodynamic radii of the protein in the presence and absence of **Cef** were measured using dynamic light scattering (Fig. 10). Computational calculations were carried out as described in the methods (Fig. 11, Fig. 12, Table 1, Table 2, Table 3).

**Table 1:** Calculated pK_a_ values for acid-range titratable residues in HEWL. Values derived from 50 ns pH-REMD simulations in the present work are shown along with literature computational (calculated on 150 ns pH-REMD simulations) and experimental values.

Residue	Calculated pK_a_ over 50 ns pH-REMD^a^	Calculated pK_a_ over 150 ns pH-REMD^b^	Experimental p*K*_a_
Glu7	3.26 (±0.17)	3.37	2.6
His15	5.71 (±0.22)	6.38	5.5
Asp18	2.93 (±0.03)	2.83	2.8
Glu35	5.95 (±0.06)	6.27	6.1
Asp48	2.62 (±0.17)	2.31	1.4
Asp52	2.42 (±0.18)	2.24	3.6
Asp66	2.46 (±0.16)	1.87	1.2
Asp87	2.74 (±0.27)	2.02	2.2
Asp101	4.17 (±0.07)	4.36	4.5
Asp119	1.96 (±0.17)	1.53	3.5

a averaged values over the whole simulated 0.8–7.8 pH range, with intervals of 1 pH unit.

b Ref. [7].

**Table 2:** Main interactions and calculated binding free energy (Δ*G*_b_) in models of the HEWL-**Cef** complexes at different pHs. “*q*” field contains the net ligand charge. Protein residues are considered interacting with **Cef** when at least one atom of the residue is within 5 Å of at least one ligand atom. “Special interactions” field includes H-bond (HB, normal style); HB reinforced by ionic interactions (**HB-II**, **bold**); π stacking (italics); positive charge-π interaction (underlined italics). NHbb and CObb indicate backbone peptide hydrogen and oxygen atoms, respectively. The SEM for ΔG_b_ values is reported.

pH	Complex^a^	*q* (a.u.)	HEWL residues interacting with **Cef**	Special interactions (HB/**HB-II**/*stacking/pos.charge-π*)	Δ*G*_b_ (kcal mol^−1^)
6.8	CED1	−2	Y20-R21, W62-W63, R73, L75, K96-K97-I98, S100-D101, N103	Y20/hydroxytriazinone	−7.18±0.012
				**R21/hydroxytriazinone**	
				**K97/hydroxytriazinone**	
				**K97/carboxylate**	
				*R73/aminothiazole*	
6.8	CED2	−2	E35, L56-Q57, W62-W63, L75, I98, N103, N106-A107-W108-V109, R112	W63/oxime	−6.74±0.011
				A107CO*bb*/amide	
				**R112/carboxylate**	
				*W108/aminothiazole*	
6.8	CED3	−2	E35, L56-Q57-I58-N59-S60-R61-W62-W63, I98, N103, N106-A107-W108-V109, R112	W63/oxime	−6.41±0.007
				A107CO*bb*/amide;	
				**R112/carboxylate;**	
6.8	CED4	−2	W62-W63, R73, L75, D101-G102-N103, N106-A107, V109, R112	R73/amide	−6.22±0.013
				D101CO*bb*/aminothiazole	
				**R112/hydroxytriazinone**	
2.8	CEN1	0	F34-G35-S36, N44, N46, D48, S50, D52, L56-Q57-I58-N59, R61-W62-W63, I98, A107-W108-V109-A110-W111	GLH35/lactam	−6.87±0.010
				A110NH*bb*/lactam	
2.8	CEP1	+1	N46, D48, S50, D52, N59, R61-W62-W63, A73, I98, D101-G102-N103-G104, A107-W108-V109	D48/hydroxytriazinone	−6.75±0.0091
				R61/hydroxytriazinone	
				W63/carboxylic group	
				N103NH*bb*/carboxylic group	
				N103NH*bb*/lactam	
2.8	CEP2	+1	N44, N46-T47-D48-G49-S50, D52, Q57-I58-N59, W62-W63, R73, L75, N103, A107-W108-V109, R112	D52/carboxyl	−6.73±0.0086
				W63/amide	
2.8	CEP3	+1	N59, W62-W63, R73, L75, S100-D101-G102-N103, A107	W63/hydroxytriazinone	−6.40±0.011
				D101CO*bb*/Amide	
2.8	CEP4	+1	I58-N59, R61-W62-W63, L75, K97, S100-D101-G102-N103, N106-A107-W108	W63/hydroxytriazinone	−6.36±0.014

a CED: aminothiazole ring neutral, both carboxylic and hydroxytriazinone groups negatively-charged; CEN: aminothiazole ring positively-charged, hydroxytriazinone group neutral, carboxylic group negatively-charged; CEP: aminothiazole ring positively-charged, both hydroxytriazinone group and carboxylic group neutral.

**Table 3:** Main interactions and calculated binding free energy (ΔG_b_) in models of the HL-**Cef** complexes at neutral pH. “*q*” field contains the net ligand charge. Protein residues are considered interacting with **Cef** when at least one atom of the residue is within 5 Å of at least one ligand atom. “Special interactions” field includes H-bond (HB, normal style); HB reinforced by ionic interactions (**HB-II**, **bold**); π stacking (italics); positive charge-π interaction (underlined italics). NHbb and CObb indicate backbone peptide hydrogen and oxygen atoms, respectively. The SEM for ΔG_b_ values is reported.

pH	Complex^a^	*q* (a.u.)	HL residues interacting with **Cef**	Special interactions (HB/**HB-II**/*stacking/pos.charge-π*)	ΔG_b_ (kcal mol^−1^)
6.8	CED1	−2	D53, F57-Q58-I59-N60, Y63-W64, V99, R107-A108-W109-V110, R113	W64/oxime	−7.13±0.011
				W64/aminothiazole	
				**R113/hydroxytriazinone**	
				*R107/hydroxytriazinone*	
6.8	CED2	−2	L31, E35, F57-Q58-I59, Y63-W64, V99, D102, Q104, R107-A108-W109-V110, R113	W64/oxime	−6.63±0.057
				A108CO*bb*/amide	
				**R113/carboxylate**	
				R113/lactam	
				*W109/aminothiazole*	

a CED: aminothiazole ring neutral, both carboxylic and hydroxytriazinone groups negatively-charged.

## 2 Experimental design, materials and methods

### 2.1 Synchrotron radiation circular dichroism (SRCD)

HEWL, recombinant HL and **Cef** were purchased from Sigma-Aldrich and used without any further purification. Proteins (0.5 mg/mL) were dissolved either in 20 mM phosphate buffer, pH 6.8, or in 70 mM glycine-HCl/80 mM NaCl buffer, pH 2.7. **Cef** stock solutions were prepared in the same buffers. Sample concentrations were determined by UV–vis spectroscopy. SRCD spectra from 180 to 260 nm were collected at Diamond B23 beamline module end-station B using integration time of 1 s, 1 nm digital resolution, 39 nm/min scan speed and 1.8 or 1.2 nm bandwidth according to the experiments. Spectra were measured using Suprasil cell (Hellma Ltd.) with 0.02 cm path length filled with 60 µL of solution. Thermal stability was monitored in the 5–90 °C temperature range at 5 °C increments with 5 min equilibration time using Quantum Peltier temperature controller. Protein UV photo-denaturation was investigated measuring twenty-five consecutive repeated scans for each sample. SRCD spectra were processed and analyzed using CD Apps software [6].

### 2.2 Secondary structure estimations (SSE)

SSE made from SRCD data collected at Diamond Light Source through CDApps software [6] using the CONTILL [6] algorithm.

### 2.3 Fluorescence spectroscopy

Fluorescence emission spectra were recorded on a Perkin-Elmer LS-50B spectrofluorimeter using emission and excitation slit widths of 2.5 nm, at 25 °C, subtracting the buffer background and correcting for dilution. Excitation wavelength was set to 295 nm [4] and the emission wavelengths were scanned from 305 nm to 475 nm in 1 nm increments. The fluorescence intensity were corrected for absorption of exciting light and reabsorption of emitted light [8], while the Trp fluorescence quenching as well as the **Cef** affinity were examined by the Stern-Volmer equation and a nonlinear regression respectively (see Material and Methods in accompanying article [1]).

### 2.4 in vivo studies

in vivo studies were conducted as described in Ref. [2].

### 2.5 Gel electrophoresis

For Native PAGE, samples were dissolved in 70 mM Gly-HCl/80 mM NaCl buffer, pH 2.7 or in 20 mM phosphate buffer, pH 6.8 and run through Native PAGE gel (gel composition: 10% acrylamide; 0.001% (v/v) TEMED; 0.004% (v/v) APS 25% (w/v)) at constant 20 mA for 2 h, in a Mini Protean 2 apparatus (BioRad, Hercules CA USA), in 43 mM imidazole and 35 mM HEPES as running buffers. For SDS PAGE samples were dissolved in 70 mM Gly-HCl/80 mM NaCl buffer, or in 20 mM phosphate buffer, pH 6.8. at constant 20 mA for 2 h, gel composition: 10% acrylamide; 0.001% (v/v) TEMED; 0.004% (v/v) APS 25% (w/v)s. BleElf Prestained Protein Marker (Jena Bioscience, Jena, Germany) was used as standard.

### 2.6 Dynamic light scattering

Solutions of HL 0.5 mg/mL in Gly-HCl saline buffer, at either pH 2.7 or 6.8, was prepared with 0.03% NaN_3_ to avoid any bacterial growth. Solutions were filtered through 0.1 μm filters to remove any aggregated material, and then were split in two Eppendorf microcentrifuge tubes. **Cef** was dissolved in the same buffer and added to one tube for each pH to achieve a final **Cef**/HEWL molar ratio 2:1, in the other tube the same buffer volume was added. DLS measurements were taken during at each temperature interval between 20 and 90 °C in 5 °C increments, and a final measurement taken at 20 °C post-thermal denaturation.

### 2.7 Computational methods

The computational methods used for data production are described in full detail in the accompanying article [1]. Briefly, ligand starting geometry and partial charges were obtained with the RESP procedure [9], using Ghemical 2.99.2 [10] and GAMESS [11] programs. Docking studies were performed with AutoDock 4.2 [12]. Amber16 package [13], with the ff14SB version of AMBER force field (FF) [14] for protein and, eventually, gaff FF [19] for the ligand was used for standard molecular dynamics (MD) simulations of Lysozyme-Cef complexes and constant pH-Replica Exchange MD (pH-REMD) of protein alone. Sequence alignments were obtained with UCSF Chimera program [15].
