# Supplementary material for: Spectroscopy data of ceftriaxone-lysozyme interaction and computational studies
Source: Data Brief. 2018 Apr 30;18:1808–18. doi: 10.1016/j.dib.2018.04.079 (PMC5998221; doi:10.1016/j.dib.2018.04.079)
Supplement: Supplementary file 1 — Supplementary material [file mmc1.docx]

**Conflict of interest
The authors declare no competing financial interest.**
